# Effects of consuming a high carbohydrate diet after eight weeks of exposure to a ketogenic diet

**DOI:** 10.1186/1743-7075-6-46

**Published:** 2009-11-19

**Authors:** Mary Ann Honors, Brandon M Davenport, Kimberly P Kinzig

**Affiliations:** 1 Department of Psychological Sciences, Ingestive Behavior Research Center, Purdue University, West Lafayette, IN, 47907, USA

## Abstract

**Background:**

Ketogenic diets have been utilized for weight loss and improvement in metabolic parameters. The present experiments examined the effects of returning to a chow diet after prolonged ingestion of a ketogenic diet.

**Methods:**

Rats were maintained on chow (CH) or a ketogenic diet (KD) for 8 weeks, after which the KD rats were given access to chow only (KD:CH) for 8 additional weeks. Caloric intake, body weight, and plasma leptin, insulin and ghrelin were measured before and after the dietary switch.

**Results:**

After 8 weeks of consuming a ketogenic diet, KD rats had increased adiposity and plasma leptin levels, and reduced insulin, as compared to CH controls. One week after the diet switch, fat pad weight and leptin levels remained elevated, and were normalized to CH controls within 8 weeks of the dietary switch. Switching from KD to chow induced a transient hypophagia, such that KD:CH rats consumed significantly fewer calories during the first week after the dietary switch, as compared to calories consumed by CH rats. This hypophagia was despite significantly increased plasma ghrelin in KD:CH rats. Finally, KD:CH rats developed hyperphagia over time, and during weeks 6-8 after the diet switch consumed significantly more calories per day than did CH-fed controls and gained more weight than CH-fed controls.

**Conclusion:**

Collectively, these data demonstrate that returning to a carbohydrate-based diet after a period of consuming a ketogenic diet has post-diet effects on caloric intake, body weight gain, and insulin levels.

## Introduction

According to the Behavioral Risk Factor Surveillance System survey conducted by the Centers for Disease Control in 2000, approximately one third of adults in the United States attempt to lose weight and another one third attempting to maintain their current weight [1]. These data imply that approximately 2 out of every 3 American adults are employing an active dietary strategy. Among such strategies are diets that control for macronutrient content, such as low-carbohydrate, ketogenic diets.

A ketogenic diet derives the majority of calories from fat and protein sources, with very limited calories from carbohydrate sources. Recent evidence indicates that dietary fat intake, as a percentage of total calories, decreased between 1971 and 2000 in both men and women, while the rate of obesity increased from 14.5% to 30.9% during this same period of time. Contrastingly, dietary carbohydrate intake has increased significantly [2,3]. In humans, ketogenic and/or low carbohydrate diets have been demonstrated to be effective in the short-term (6 months or 1 year) for weight loss [4-6]. Furthermore, it has been demonstrated that restriction of dietary carbohydrates results in positive effects on cardiovascular parameters, favorably affects body adiposity, and improves features of metabolic syndrome [7-11].

In rats, it has been demonstrated that the effects of a ketogenic diet on body weight and caloric intake differ from those of a high-carbohydrate, high-fat diet. When consuming the latter, rats gain significantly more weight than do chow-fed controls [12,13]. In contrast, Thenen and Mayer established that rats consuming a ketogenic diet (2% of calories from carbohydrates, 50% fat, and 48% protein) gained weight in a similar manner as those fed a control diet [14]. Since these original experiments, others have confirmed this phenomenon with similar diets [15-18]. After seven weeks, rats fed a ketogenic diet (5% of total kcal from carbohydrate sources, 80% from fat, and 15% from protein) gained significantly less body weight than rats fed a high fat, high carbohydrate diet (25% of total kcal from carbohydrate sources, 60% from fat, and 15% from protein) (13). Contributing to the increase in body weight gain exhibited by rats fed a high fat, high carbohydrate diet, these rats consume more calories than chow-fed controls. In contrast, ketogenic diet-fed rats consume fewer calories than high fat-fed rats, with mean weekly caloric intake that is comparable to that of chow-fed controls [12,13,15,17]. Despite the elevated fat content of the ketogenic diet, it appears that a reduction in carbohydrate intake attenuates the effects of dietary fat on body weight gain and caloric intake.

Here we sought to examine the effects of returning to a high carbohydrate diet after 8 weeks of consuming a ketogenic diet. Following 8 weeks of consuming on a ketogenic diet, rats were fed a standard chow diet for an additional 8 weeks, with body weight, caloric intake, epididymal fat pad weight, and endocrine profiles measured before and periodically after the diet switch. It was hypothesized that resumption of a chow diet would result in hyperphagia and increased body weight gain, as compared to rats that were only given access to chow throughout the experiment.

## Materials and methods

Male Long Evans rats (Harlan, Indianapolis, IN), weighing 225-250 g at the start of the experiment, were individually housed in hanging wire mesh cages in a climate-controlled room with a 12:12 h light: dark cycle (lights off at 13:00). Rats were allowed *ad libitum *access to standard laboratory chow (2018, Harlan Teklad, Indianapolis, IN) for one week, during which time the rats acclimated to the laboratory environment. All procedures were approved by the Purdue Animal Care and Use Committee (PACUC).

Following acclimation to the laboratory environment, rats were weight matched and assigned to one of two dietary groups: chow (CH), or ketogenic diet (KD). Chow (2018, Harlan Teklad, Indianapolis, IN) was formulated such that 60% of total kilocalories were from carbohydrate sources, 17% from fat, and 23% from protein. In contrast, the KD contained 5% of total kilocalories from carbohydrate sources, 80% from fat, and 15% from protein (Diet 06040602, Research Diets Inc., New Brunswick, NJ, Table 1). All rats were allowed *ad libitum *access to their assigned diet for 1, 4 or 8 weeks. Body weights and caloric intake were recorded daily, with food spillage collected and factored into calculations of daily caloric intake.

**Table 1:** Components of the ketogenic diet (Research Diets, D06040601)

	Chow Diet	Ketogenic Diet
	Kcal %	Kcal %
**Protein**	23	15
**Carbohydrate**	60	5
**Fat**	17	80

**Total**	100	100
**Kcal/g metabolizable energy**	3.3	6.1

After 1, 4 or 8 weeks of consuming the assigned diet, rats were sacrificed (n = 6-7 rats per dietary group at each time point). Food was removed from the cage 3 h prior to the onset of the dark cycle, and rats were rapidly decapitated under ether inhalation anesthesia 1 h later. Trunk blood was collected in K+EDTA treated vacutainer tubes, and a small sample (approximately 50 μL) of blood was removed to test levels of β-hydroxybutyrate using a StatSite meter (Stanbio Laboratory, Boerne, TX) and StatSite Blood Ketone Test Cards (Stanbio Laboratory, Boerne, TX). Levels of β-hydroxybutyrate in blood were measured as an indication of whether an animal was in a state of ketosis, as β-hydroxybutyrate is the ketone body produced in the highest amount during ketosis [19]. The remaining blood from each rat was then centrifuged at 2000 rpm at 4°C for 15 minutes. Plasma was aliquotted into chilled 1.5 mL eppendorf tubes and stored at -80°C until processing for analysis of plasma insulin, leptin, and ghrelin levels. In addition, the epididymal fat pads of each rat were removed and weighed at the time of sacrifice as a measure of body adiposity. In male rats, epididymal fat pad weight correlates positively and strongly (*r *= 0.91) with body weight across the growth curve from approximately 180 to 800 g [20].

Using a second cohort of rats, the effects of returning to a chow diet after 8 weeks of consuming KD were examined. For this experiment, male Long Evans rats (Harlan, Indianapolis, IN), weighing 225-250 g at the start of the experiment, served as subjects. Following acclimation to the laboratory environment, rats were weight matched and placed on chow or the ketogenic diet (n = 25 per diet group). Body weight and caloric intake were recorded daily, with food spillage collected and factored into calculations of daily caloric intake. Rats were maintained on their assigned diet for 8 weeks and then the KD group was switched to chow (KD:CH) for an additional 1, 4, or 8 weeks. CH rats were maintained on chow for the entire 16-week duration of the experiment.

Rats from each dietary group were sacrificed 1, 4, and 8 weeks following the switch to chow (n = 6-7 rats per dietary group at each time point). Rats were sacrificed as described above, and plasma was aliquotted into chilled 1.5 mL eppendorf tubes and stored at -80°C until processing. In addition, the epididymal fat pads of each rat were removed and weighed at the time of sacrifice as a measure of body adiposity.

### Radioimmunoassay

Plasma insulin, leptin, and ghrelin were determined using commercial radioimmunoassay (RIA) kits (Linco Research, St. Charles, MO). All samples were run in duplicate and per manufacturer's instructions. The plasma insulin RIA kit had upper and lower detection limits of 0.1 ng/mL and 10 ng/mL, respectively. The plasma leptin RIA was run with upper and lower detection limits of 0.5 ng/mL and 50 ng/mL, respectively. The ghrelin RIA kit had upper and lower detection limits of 100 pg/mL and 10,000 pg/mL, respectively. Unknown concentrations of hormones in plasma samples were calculated based on standard curves generated for each kit.

### Statistical Analyses

Data were analyzed to examine the effects of maintenance on LC on body weight, caloric intake, body adiposity, blood β-hydroxybutyrate levels, and endocrine profiles. All statistical analyses were performed using SPSS Statistics Software, version 17.0. Significance was determined at an alpha level of 0.05.

Caloric intake and change in body weight data were analyzed by repeated measures two-way analysis of variance (ANOVA). Significant main effects and interactions were examined further via post-hoc Tukey HSD tests. Epididymal fat pad weight, blood β-hydroxybutyrate, and hormone concentrations were analyzed by one-way ANOVA to determine differences between diet groups. All data are represented as mean ± standard error of the mean (SEM).

## Results

### Body weight change and caloric intake in response to consuming chow, a ketogenic diet, and switching from a ketogenic diet to chow

**Figure 1:**
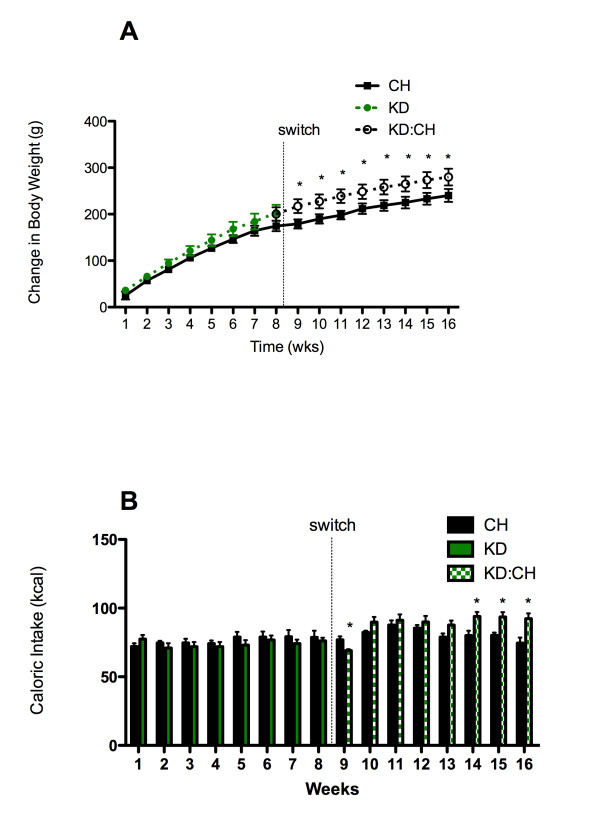
**Body weight change and caloric intake**. Body weight (A) increased similarly in KD and CH over time until KD rats were switched to CH. After the switch, the KD:CH group had a significantly greater change in body weight. Mean weekly caloric intake was not different between dietary groups prior to the dietary switch (B). KD:CH rats demonstrated a transient decrease in caloric intake and subsequent hyperphagia, as compared to CH rats, for the final 3 weeks of the experiment. Data are represented as mean ± SEM. * denotes a statistically significant difference from CH values.

The effects of diet and time on body weight change after 8 weeks of consuming KD and then switching to a carbohydrate-based diet were assessed daily and are shown in Figure 1A. During the period in which rats consumed chow (n = 7) or KD (n = 7), rats in both dietary groups exhibited similar patterns of body weight gain. Prior to the dietary switch (weeks 1-8) there were no differences in the amount if weight gained by CH or KD rats. After the dietary switch (KD:CH), cumulative body weight gain was significantly greater in the KD:CH group than in the CH group at weeks 9-16 (p < 0.05 for each week).

Caloric intake is shown in Figure 1B. During the first 8 weeks of the experiment, there were no differences in mean caloric intake (by week) between dietary groups. Upon switching from KD to CH, the KD:CH group exhibited a transient decrease in caloric intake such that KD:CH intake at week 9 of the study was significantly lower than CH intake (p < 0.05). This increase was attenuated in the second week after the dietary switch and ultimately reversed such that KD:CH rats consumed significantly more calories than did CH rats in weeks 14, 15, and 16 after the diet switch (p < 0.05 for weeks 14, 15, and 16).

**Figure 2:**
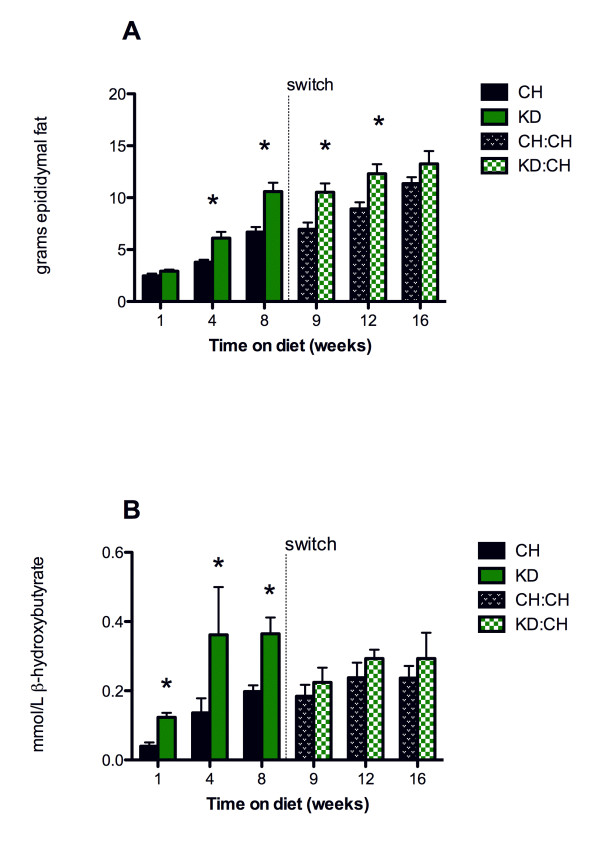
**Fat pad weight and β-hydroxybutyrate levels after 8 weeks of maintenance on a ketogenic diet or chow**. (A) Epididymal fat pad weight was significantly greater in KD rats after 4 and 8 weeks of diet exposure (p < 0.05). This difference was no longer present 8 weeks after the diet switch (experimental week 16). The level of *β*-hydroxybutyrate present in blood (B) was significantly increased (p < 0.01) after one week of exposure to the ketogenic diet, but not after rats were switched from the ketogenic diet to chow (week 9). Data are represented as mean ± SEM. * denotes a statistically significant difference from CH values.

As shown in Figure 2A, KD rats had significantly more epididymal fat than CH controls by the time they had been consuming KD for 4 weeks (KD: 6.11 ± 0.59 and CH: 3.80 ± 0.23 g, p < 0.05), with a greater difference after 8 weeks (KD: 10.60 ± 0.92 and CH: 6.89 ± 0.53 g, p < 0.001). Epididymal fat pad weights remained elevated at weeks 9 and 12 (1 and 4 weeks after the dietary switch) as compared to CH rats (KD:CH week 9: 10.53 ± 0.84, CH week 12: 6.96 ± 0.65 g, p < 0.05). This difference persisted to week 12, and was no longer present at week 16. Interestingly, fat pad weights did not change statistically in KD:CH rats after switching to chow, and the abolition of the difference between KD:CH and CH rats was due to a significant increase in CH fat pad weights between weeks 9 and 16 (p < 0.05). β-hydroxybutyrate levels (Figure 2B) were significantly elevated in KD rats at all time points, as compared to CH controls, (p < 0.01 for 1, 4, and 8 weeks). β-hydroxybutyrate levels ranged from 0.01 to 0.76 mmol/l. There were no differences between dietary groups after the KD group was switched to CH.

### Endocrine effects following intake of chow, a ketogenic diet, and switching from a ketogenic diet to chow

**Figure 3:**
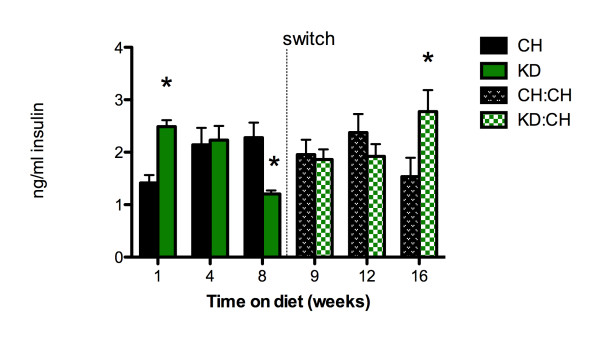
**Plasma insulin**. (A) Plasma insulin was significantly lower in rats maintained on KD after 8 weeks (p < 0.05). Eight week after switching to chow (experimental week 16), KD:CH insulin was significantly elevated as compared to insulin levels of CH controls that had never consumed KD (p < 0.05). Data are represented as mean ± SEM. * denotes a statistically significant difference from CH values.

After 1 week of consuming the ketogenic diet, KD rats had significantly higher plasma insulin levels than did CH controls (2.49 ± 0.12 and 1.42 ± 0.15 ng/mL insulin, p < 0.05, Figure 3). There were no differences between dietary groups at week 4. After 8 weeks of consuming KD, insulin was significantly lower in the KD group than in the CH group (1.18 ± 0.12 and 2.50 ± 0.59 ng/mL, respectively, p < 0.05). There were no differences in plasma insulin levels between KD:CH and CH groups at weeks 9 or 12. However, there was a significant increase in plasma insulin 8 weeks after the switch in KD:CH rats (experimental week 16). At 2.78 ± 0.41 ng/mL, KD:CH insulin levels were significantly greater than CH insulin at week 16 (1.53 ± 0.36 ng/mL, p < 0.05) and elevated as compared to KD:CH levels one week after the switch (2.06 ± 0.26 ng/mL, p < 0.05).

**Figure 4:**
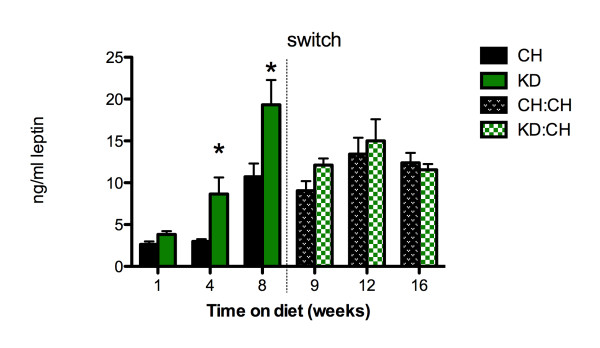
**Plasma leptin**. Plasma leptin levels were elevated after 4 and 8 weeks of KD exposure (p < 0.05), and remained increased 1 week after switching to chow in the KD:CH group. Data are represented as mean ± SEM. * denotes a statistically significant difference from CH values.

Plasma leptin levels (Figure 4) were significantly increased in KD rats as compared to CH rats at 4 weeks (8.65 ± 1.99 and 2.99 ± 0.26 ng/mL, respectively, p < 0.05), and remained elevated at 8 weeks (19.32 ± 2.97 and 10.72 ± 1.58 ng/mL leptin, respectively, p < 0.05). Consistent with the elevated fat pad weight in KD:CH rats 1 week after the dietary switch (experimental week 9), plasma leptin levels remained significantly elevated in KD:CH, as compared to CH leptin (KD:CH: 12.11 ± 0.8 and CH: 9.09 ± 1.16 ng/mL, p < 0.05). The differences in plasma leptin levels were attenuated by week 12, such that there were no differences between dietary groups. Similar to the effects of switching from the ketogenic diet to chow on epididymal fat pad weight, the level of leptin in KD:CH rats did not change during the 8 weeks after the diet switch, whereas leptin levels in CH rats increased significantly 4 weeks after the KD:CH rats underwent the diet switch (experimental week 12), as compared to leptin at baseline in this group (p < 0.05).

**Figure 5:**
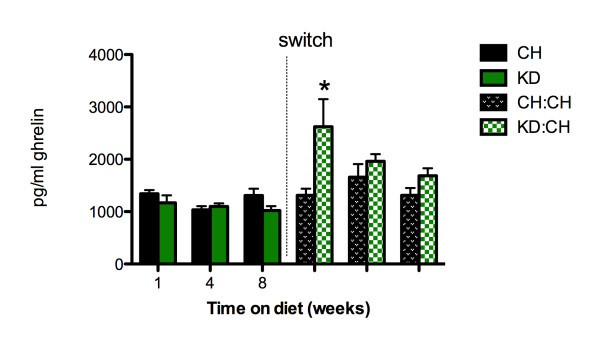
**Plasma ghrelin**. There were no differences between dietary groups with regard to plasma ghrelin levels prior to the dietary switch. Plasma ghrelin levels were significantly increased in KD:CH (p < 0.05) rats one week after switching from the ketogenic diet to chow, as compared to CH rats at this time point. This effect was only present at this time point and in this dietary group. Data are represented as mean ± SEM. * denotes a statistically significant difference from CH values.

Analysis of plasma ghrelin levels after 8 weeks of KD (Figure 5) determined that there were no diet-related differences between dietary groups at any time point prior to the dietary switch. Comparison of plasma ghrelin levels revealed that one week after switching from the ketogenic diet to chow, KD:CH ghrelin was significantly higher than it was in CH rats (KD:CH: 3115.49 ± 532.4 and CH: 1865.63 ± 151.18 pg/mL, p < 0.05) and in KD rats that were maintained on KD for 8 weeks and not switched to CH (p < 0.05). This difference was only present one week after the dietary switch.

## Discussion

The purposes of the present experiments were to examine (A) the time course of endocrine changes with exposure to a ketogenic diet and (B) the effects of returning to a high carbohydrate diet after a period of consuming a ketogenic diet. We demonstrate that immediately following the discontinuation of the ketogenic diet, rats developed a brief period of hypophagia that was accompanied by a transient increase in plasma ghrelin levels, and a subsequent hyperphagia that developed 6 weeks after the dietary change and persisted through the end of the study. The increased chow intake was accompanied by significantly greater increases in body weight after the dietary switch, as compared to rats consuming chow for the entirety of the experiment. Whereas KD rats had significantly increased fat pad weights and plasma leptin levels as compared to CH rats, resuming the chow diet prevented a further increase in adiposity and leptin over time. Rats that consumed chow for the entirety of the study increased fat pad weight and leptin to resemble those of KD:CH rats by the end of the study. In addition, plasma insulin levels in KD:CH rats were not different from CH rats one week after returning to the chow diet, although it was significantly increased after 8 weeks of consuming chow after the ketogenic diet.

Previous studies in which rats with a history of consuming a ketogenic diet are switched to a high-carbohydrate, low-fat chow diet have observed an initial, short-term decrease in caloric intake following the diet switch [15,16,21,22]. This effect has been found to last a short duration, with caloric intake returning to pre-switch levels after one week or less [15,16]. In the present experiment, KD:CH rats initially decreased caloric intake during the first week after changing diets. This difference was transient and dissipated by the second week after the dietary change. It is likely that the increased ghrelin measured in this group one week after the dietary switch is a reflection of the transiently decreased caloric intake. Following the early hypophagia, caloric intake increased over time in KD:CH-fed rats after 6 weeks of consuming chow, as compared to caloric intake by CH rats.

Levels of leptin in plasma circulate in proportion to levels of body adiposity [23]. In the present experiment, KD rats exhibited increased plasma leptin levels, as compared to CH. These data are in accordance with previous observations that chronic consumption of a ketogenic diet induces an increase in this anorexigenic signal [17,24]. As was the case with adiposity levels following the switch to CH, plasma leptin levels of KD:CH rats remained similar to levels prior to the diet switch. These results are in agreement with prior studies in which the short-term effects of a low- to high-carbohydrate diet switch were examined. Del Prete and colleagues have previously observed that plasma leptin levels remained steady two days after a similar diet switch [16]. In contrast, CH controls exhibited a significant increase in plasma leptin levels over time, and ultimately increased to the level of KD:CH leptin by the end of the study.

Ingestion of a ketogenic diet, at least in the rat, produces a unique endocrine state. While there is generally a positive correlation between levels of leptin and insulin [25], consuming a ketogenic diet alters this relationship such that leptin is exceedingly high while insulin levels are depressed. In this experiment, as in previous studies [17,26], KD rats had lower plasma insulin levels than did CH controls despite increased levels of body adiposity. Although we report that insulin was significantly increased in KD rats after one week on the diet, it is likely that this was due to low levels of insulin in the CH rats at this time point, rather than a diet-induced rise in insulin from consuming a ketogenic diet. Interestingly, a significant decrease in plasma insulin was not measured until rats had been consuming KD for 8 weeks. Levels of circulating insulin are known to fall during other states of ketosis, such as starvation [27]. In addition, injections of an insulin anti-serum have been shown to increase concentrations of ketone bodies in blood [28]. Here, although levels of blood β-hydroxybutyrate were significantly elevated within one week of consuming the ketogenic diet, insulin was not decreased until week 8. The relationship between ketosis and insulin requires further investigation. It may be the case that over time, the process of ketosis has an inhibitory effect on insulin production and result in a dissociation between adiposity and plasma insulin levels.

In these experiments, returning to a carbohydrate diet after 8 weeks of consuming a ketogenic diet initially resulted in normalization of KD:CH insulin levels, compared to CH controls. After 8 weeks of consuming chow, the KD:CH rats had significantly elevated plasma insulin levels. It is likely that the increase in insulin is associated with increased caloric intake by KD:CH rats at this time point, as this increase corresponds with the increase in caloric intake exhibited by these rats in the last 3 weeks of the study.

Collectively, the present data demonstrate that following the switch from a ketogenic diet to one that is high in carbohydrates, rats gained more weight than did rats that had never consumed the ketogenic diet. Levels of epididymal fat were maintained at pre-switch levels throughout the chow-feeding period, although rats that underwent the dietary switch increased caloric intake over time and became hyperinsulinemic as compared to CH-fed controls. Whether these effects are observed in humans that choose to return to a high-carbohydrate diet after periods of consuming diets low in carbohydrates is currently unknown, however the current data suggest that continued maintenance on a low carbohydrate diet may play a role in prevention of increased body weight and caloric intake.

## Declaration of competing interests

The authors declare that they have no competing interests.

## Authors' contributions

MH designed the experiments, performed the immunoassays and statistical analyses, and helped to draft the manuscript. BD assisted with all aspects of data collection and analysis. KK participated in the study design and coordination and assisted in drafting the manuscript. All authors read and approved the final manuscript.
